# Gender-Based Differences and Barriers in Skin Protection Behaviors in Melanoma Survivors

**DOI:** 10.1155/2016/3874572

**Published:** 2016-08-25

**Authors:** Jeffrey Chen, Johnny Shih, Andrew Tran, Aaron Mullane, Christina Thomas, Nail Aydin, Subhasis Misra

**Affiliations:** ^1^Texas Tech University Health Sciences Center, 1400 S. Coulter Street, Amarillo, TX 79106, USA; ^2^Department of Surgery, Texas Tech University Health Sciences Center, 1400 S. Coulter Street, Amarillo, TX 79106, USA

## Abstract

*Purpose*. Skin protection behaviors and environmental exposure play a crucial role in the development and subsequent management of melanoma. This study investigates gender-based differences in skin protection behaviors after melanoma treatment.* Methods*. Patients diagnosed and surgically treated for cutaneous melanomas over the last six years in a geographically high risk area were surveyed over telephone using a standardized script.* Results*. Of 150 survey results obtained, there were 82 males and 68 females. Overall, 87% of participants reported skin self-examination for abnormal markings more often and 94% reported wearing skin protective clothing more often, with females being more than males. Females limited outdoor activity more often than males, 79% to 54%, *p* < 0.05. When outside, females sought shade more often than males, 75% to 56%, *p* < 0.05. However, males wore a wide brim hat more often than females, 52% to 28%, *p* < 0.05. Interestingly, 60% of participants reported wearing SPF 30 sunscreen less often, *p* < 0.05.* Conclusion*. Larger percentage of females adopted behavioral changes to prevent future melanoma. Those living in high risk areas and with outdoor occupations need particular attention to skin care. Population based screening should be adopted to deal with this rising public health crisis.

## 1. Introduction

Skin cancer is considered the most common cancer in the United States. Melanoma is a malignancy of melanocytes, pigment-producing cells that reside in the basal epidermal layer. Melanoma accounts for less than 2% of skin cancers but accounts for most skin cancer related deaths [1]. The incidence of melanoma has been increasing for the past 30 years [2]. In 2015 alone, there were approximately 74,000 newly diagnosed cases, 5% of which were from Texas [1, 3]. The Texas Panhandle has one of the highest incidence rates in the state with 21.5 per 100,000 persons compared to the state average of 14.1 per 100,000 [4]. The Texas Panhandle also claims melanoma death rates nearly twice the national average [5, 6]. The national incidence and mortality of melanoma vary by gender, accounting for the 5th and 7th most common cancers in men and women, respectively [7]. In 2015, 67% of melanoma deaths occurred in men [1].

Risk factors that increase the risk of developing melanoma include “blue or green eyes, light colored skin, dysplastic nevi, abnormal moles, blistering sunburns, excessive UV exposure, psoralen therapy with UVA light, immunosuppression, and habitation closer to the equator or at higher elevations” [8–12]. Studies have also isolated six high risk factors that independently increase the risk of getting melanoma. They are “actinic keratosis, blond or red hair, family history of melanoma, upper back freckles, three or more blistering sunburns prior to age 20, and three or more years of outdoor occupation as a teenager” [13–15]. Individuals with one or two of the risk factors have a tripled risk of developing melanoma compared to the rest of the population, and patients with previous melanoma have a ninefold increase in risk of developing subsequent melanoma compared to the rest of the population [9, 16]. Thus, skin protection behaviors and environmental exposure play a crucial role in the development and subsequent management of melanoma. Recommendations to reduce the risks of melanoma deaths and recurrence include routine self-examinations for suspicious lesions, sun avoidance, use of sunscreen, and protective clothing [17]. However, not much research is available on skin protection behaviors after melanoma treatment, especially between genders. This study aims to investigate gender differences in skin protection behaviors after melanoma treatment.

## 2. Methods

We looked at a representative sample of cutaneous melanoma cases diagnosed and surgically treated in the Texas Panhandle, a geographically high risk area. All patients were treated by surgical oncologists between 2008 and 2015. Inclusion was limited to living patients of 18 years or older with biopsy-proven and surgically treated melanoma. Telephone surveys lasting 8 to 10 minutes were conducted using a standardized script with three main categories consisting of health maintenance, skin protection, and sun avoidance as follows.

*Telephone Survey Questions*

*Section I: Health Maintenance*

1. How often in the past year have you visited any physician for a skin examination?
2. How often in the past year have you had a full body skin check by a healthcare provider?
3. How often in the past year have you self-examined your skin for abnormal markings (changing color, getting bigger, new mole) for growths?

*Section II: Skin Protection*

(4) How often do you wear a hat with a wide brim all the way around?
(5) How often do you wear long sleeved shirts?
(6) How often do you wear sunscreen of at least SPF 30
(7) How often do you wear sunglasses?
(8) How often do you wear pants that reach your ankles?

*Section III: Sun Avoidance Behavior*

(9) How often do you limit your outdoor activity?
(10) When outside, how often do you seek shade?
(11) How often do you worry about developing another case of skin cancer?
(12) How often do you wear a hat, scarf, cap, or use an umbrella?

*The Last 2 Questions Have Different Answer Choices*

(13) Since being diagnosed with melanoma, how often do you take part in outdoor activities compared to before the diagnosis?
 Significantly less, slightly less, the same, slightly more, significantly more
(14) Since being diagnosed with melanoma, how often do you worry about your melanoma compared to before the diagnosis?
 I worry a lot less, I worry slightly less, I worry about the same amount, I worry slightly more, I worry a lot more

Surveys were mailed in prepaid envelopes to those who did not respond to at least three of our phone calls. Data was evaluated and *p* values were calculated for each of the survey questions.

## 3. Results

Responses were collected from 150 of 500 contacted participants, a 30% return rate. The participants included 82 males and 68 females with ages ranging from 18 to 88 years (Table 1). Overall, 87% of participants reported skin self-examination for abnormal markings more often, with females self-examining more often than men (Table 2). Of all participants, 94% reported wearing skin protective clothing (sunglasses, pants that reach the ankles, and long sleeved shirts) more often, with females being more than males. Females limited their participation in outdoor activities after their diagnosis of melanoma to a greater degree than males, 79% to 54%, *p* < 0.05. When outside, females sought shade more often than males, 75% to 56%, *p* < 0.05. Of all participants, 81% worried more about their melanoma compared to before their diagnosis with females worrying more than males, 90% to 73%, *p* < 0.05. On the contrary, males wore wide brim hats more often than females, 52% to 28%, *p* < 0.05. Interestingly, 60% of participants reported wearing SPF 30 sunscreen less often, with 67% of males and 52% of females wearing sunscreen less often than prior to their melanoma diagnosis, *p* < 0.05. We also looked at age, separating the patients into two age groups, over 55 and under 55, but results were not statically significant.

## 4. Discussion

In 2013, the American Association for Cancer Research reported that between 1990 and 2009, melanoma death rates increased by 10.5% in men, while in the same period, melanoma death rates decreased by 9.6% in women [1, 3]. Over the past decade, studies have reported a gender disparity in the biology of melanoma, with males having an increased risk of having thicker (>2 mm) tumors, higher risk of progression, and higher risk of metastasis [18, 19]. Our study looked into what seems to be lacking in the literature, to see whether or not there is a gender-based difference in behavior after the treatment of melanoma.

Results showed that a larger percentage of females compared to males adopted behavioral changes to prevent future cutaneous melanoma. Females were more likely to self-examine their skin for abnormal markings, and previous studies have shown a correlation between self- examinations and earlier melanoma diagnosis. Females were also more likely to wear protective clothing, limit their outdoor activity, and seek shade, which would ultimately lessen their exposure to UV radiation and decrease their risk of developing blistering sunburns, both of which are risk factors for melanoma. Women also worried more about their melanoma, which may be the underlying reason for their increased emphasis on preventative measures. One finding that seemed to deviate from the pattern was that men seem to wear a wide brim hat more often than females. This could possibly be due to behavioral change, but we hypothesize that it is more likely that males have careers involving agriculture or livestock in West Texas, so they are more likely to be outdoors for longer periods of time. Agriculture makes up the largest sector of the Texas Panhandle economy, and this predominately male workforce engaged in outdoor occupations including farming, ranching, and construction have a 6–8x higher UV exposure compared to workers in indoor occupations [20, 21].

Melanoma recurrence is higher than once thought, occurring in more than 6% of patients after 10 years, 6.8% of patients after 15 years, and 11.3% of patients after 25 years. Patients facing late recurrence were typically younger, with an average age of 41 to an average of 51 for early recurrence of melanoma [22, 23]. Studies have shown that sunscreen use decreases the incidence of sunburns. Sunburns, which can increase the likelihood of getting melanoma, are preventable, yet nearly 40% of the US population report getting sunburns each year [24, 25]. Thus, it is discouraging to find that more than half of the participants in both genders reported wearing sunscreen with greater than SPF30 less often than before their diagnosis of melanoma. We also looked at age to see if a certain age group was more or less likely to use sunscreen, but the data for two age groups, over 55 and under 55, was not statistically significant. The majority of respondents spent less time in outdoor activities after their diagnosis of melanoma. When asked about their sunscreen habits, the majority of those who spent less time outdoors believed that since they were outside for shorter periods of time, they no longer needed sunscreen. The second most popular response was that since sunscreen did not prevent melanoma in the first place, it probably was not going to prevent a recurrence.

Our results point to the need of stronger patient education on the possibility of melanoma recurrence and on proper sunscreen usage to dispel misconceptions surrounding application efficacy. Due to its preventable nature and its increasing incidence, melanoma has been labeled a public health crisis by the US Surgeon General because a person who dies from melanoma has lost on average 20 years of potential life [1, 26, 27]. While there are no national guidelines in the United States regarding skin cancer screening, developed countries around the world, such as Belgium, Germany, Australia, France, and Japan have adopted population based screening for skin cancer. Germany has seen a nearly 40% decrease in melanoma mortality as a result [28, 29]. Patient adherence to preventative measures such as sunscreen needs to be pursued more aggressively. With 67% of melanoma deaths occurring in men and with less male attention to health maintenance, skin protection, and sun avoidance after melanoma treatment, more needs to be done in terms of education and follow-up, and mass population skin cancer screening should be adopted [1–3].

## Figures and Tables

**Table 1:** Demographic characteristics.

	Number	%
*Age*		
Average	59.4	
Range	18–88	
*Gender *		
Male	82	55
Female	68	45
*Stage*		
Stage 0	38	25.3
Stage 1	91	60.7
Stage 2	7	4.7
Stage 3	4	2.7
Stage 4	0	0
Unstaged	10	6.7

**Table 2:** Results showed that a larger percentage of females compared to males adopted behavioral changes to prevent future cutaneous melanoma, with the exception of the wide brim hat. A majority of both females and males used sunscreen of at least SPF 30 less often than prior to diagnosis.

	Male (%)	Female (%)
*More often*		
Wear wide brim hat	52	28
Limit outdoor activity	54	79
Seek shade	56	75
Worry about recurrence	73	90
*Less often*		
Sunscreen SPF > 30	67	52
